# The impact of climate change on photovoltaic power potential in Southwestern Colombia

**DOI:** 10.1016/j.heliyon.2022.e11122

**Published:** 2022-10-14

**Authors:** Gabriel Narvaez, Luis Felipe Giraldo, Michael Bressan, Andres Pantoja

**Affiliations:** a Department of Electrical and Electronic Engineering, Universidad de Los Andes, Bogota, Colombia; b Department of Biomedical Engineering, Universidad de Los Andes, Bogota, Colombia; c Department of Electronic Engineering, Universidad de Nariño, Pasto, Colombia

**Keywords:** Surface solar radiation, Photovoltaic power, Solar energy, CORDEX, Colombia

## Abstract

In this paper, we present the first study of the long-term climate-change impact on photovoltaic power potential in Nariño, Colombia. In this region, more than half of the territory does not have a constant electricity supply, but it has great potential for solutions with renewable energy sources. Based on the Coordinated Regional Downscaling Experiment (CORDEX), we assess the change in photovoltaic power potential towards the end of this century, considering two climate change scenarios, one optimistic and the other pessimistic. Our results suggest that changes in photovoltaic power potential, by the end of the century, will have a maximum decrease of around 2.49% in the central zone of Nariño, with some non-affected areas, and a maximum increase of 2.52% on the southeastern side with respect to the pessimistic climate change scenario.

## 1 Introduction

**Figure 1:**
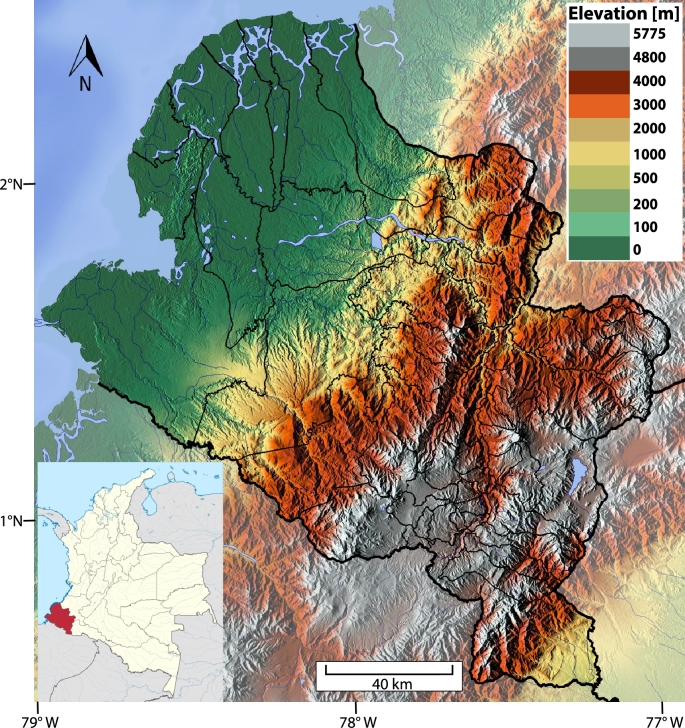
Nariño state is located in southwestern Colombia, bordering Ecuador and the Pacific Ocean. There are three physiographic regions: the Pacific region in the northwest, characterized by high temperatures, abundant rainfall, and lush vegetation; the Andean region, formed by the Andes mountain range, which is the most populated region; and the Amazonian slope in the south, which is covered by rainforests, with steep and little usable terrains.

Colombia is a country in South America in which around 52% of the territory belongs to the non-interconnected zone, involving 1,798 rural communities [1]. Only 79% of them have electricity between 1 and 6 hours per day, 17% have more than 6 hours of daily electricity supply, and the remaining 4% have no electricity supply service. From the total number of isolated communities in this situation, 33% are in Nariño ($1^{\circ }16^{\prime }59^{\prime \prime }$ North, $77^{\circ }22^{\prime }1^{\prime \prime }$ West), a region in South-west Colombia, corresponding to a half of the communities in this region. This issue makes it the region with the highest percentage of non-interconnected rural communities in Colombia [2], with a noticeable requirement of alternative energy solutions. An important characteristic of this region is its complex topography, which ranges from mountainous to maritime zones, as depicted in Fig. 1,^1^ where mountains reach approximately 5,770 meters above sea level. Installation of power lines is difficult due to wilderness areas, lack of roads, and deficient infrastructure [3], [4]. To address this problem, Nariño's Sustainable Rural Energization Plan (PERS-Nariño) [5] proposed the implementation of several renewable energy projects around the zone involving photovoltaic (PV) solutions. However, even though it is well known that climate change can affect PV solutions in the medium and long term [6], [7], there are no reported studies analyzing climate change in Nariño and its effect on the performance of PV generation systems. This situation limits the impact of such renewable energy projects, which are key for social development in Nariño.

The contribution of this work is the evaluation of the climate change impact on the PV energy production in Nariño. As it has been done to understand the impact of climate change on PV power production in regions of Europe [8], [9], China [10], and Africa [11], [12], we used climate models to study how solar irradiance, temperature, and wind speed changes will affect the solar PV potential towards the end of the century. These widely studied models predict climate changes around the world using several weather variables including different scenarios for greenhouse gas emissions at different levels of resolution [8], [10], [11], [13], [14].

In particular, we used data from the Coordinated Regional Downscaling Experiment CORDEX [15], which have been widely used to study climate change projections at a local scale [16], [17], [18] and to assess the impact of climate on the PV power potential [8], [10], [19], [20]. We have determined the regions of Nariño with the largest solar PV potential, and the mostly affected regions by climate change. This study may become a reference for future implementation of photovoltaic systems in the region, achieving a sustainable solution in the medium and long term.

This paper is organized as follows. Section 2 presents the proposed methodology, and data collection process. Then, results are presented in Section 3, followed by a discussion and conclusions in Sections 4 and 5. Finally, an appendix section describes the mathematical formulation of the PV power potential and discussion on the data validation process.

## 2 Materials and Methods

**Figure 2:**
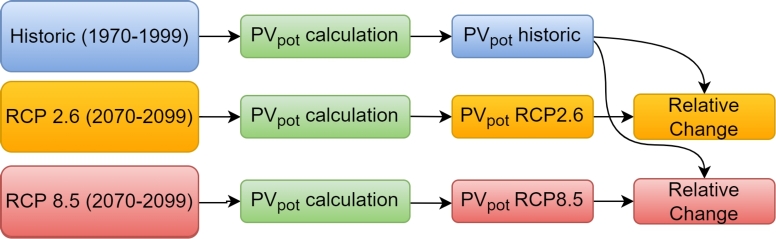
The proposed study involves estimating PV power potential (*PV*_*pot*_) at the end of the century (2070-2099) from two climate changes models (RCP8.5 and RCP2.6) and comparing them to the PV power potential in the reference period (1970-1999).

The impact of climate change on PV generation in Nariño is analyzed by comparing the difference between the PV power potential in a reference period (1970-1999) with the estimated PV power potential at the end of the century (2070-2099), following a formulation similar to the one presented in [8], [10], [14]. Fig. 2 illustrates the applied methodology. The first step included collecting data from projections of surface-downwelling shortwave radiation (RSDS), surface air temperature (TAS), and surface wind speed (WS). Then, we applied a formulation to estimate the PV power potential in the reference period and under the two climate change scenarios. Finally, we calculated the relative change of the PV power potential for each climate scenario with respect to the reference period.

The CORDEX-Coordinated Output for Regional Evaluations (CORE) provides high-resolution regional climate model (RCM) projections with greater detail and accurate representation of localized events [15]. In CORDEX-CORE, two RCMs were used to downscale four global climate models (GCMs) under two climate scenarios. Table 1 shows the GCM and RCM models available for South America [21].

**Table 1:** Overview of the analyzed CORDEX-CORE experiments. Each experiment has one historical and two scenarios (RCP2.6 and RCP8.5), spanning the periods 1970-2005 and 2070-2099 respectively. The horizontal resolution of all simulations is 0.22º in both latitude and longitude.

Forcing GCM run	RCM	
	GERICS-REMO2015	ICTP-RegCM4-7
MOHC-HadGEM2-ES	✓	✓
MPI-M-MPI-ESM-LR	✓	
MPI-M-MPI-ESM-MR		✓
NCC-NorESM1-M	✓	✓

Different studies have demonstrated the accuracy of CORDEX-CORE models in providing consistent and high-resolution regional climate change projections [22], [23]. These models have also been used to assess the impact of climate change on renewable energies [24]. These climate change models are based on representative concentration pathways (RCPs) estimating different greenhouse gas emission scenarios. The RCP2.6 scenario represents the case where the increase of global mean temperature reaches a maximum of $2^{\circ }$C by the end of the century, which is the best-case scenario. The policies required to achieve this goal include large penetration of renewable energy sources, drastic reduction of fossil fuels, and international collaboration. On the other hand, the RCP8.5 represents the worst-case scenario, where the increase of global mean temperature reaches a maximum of $4^{\circ }$C by the end of the century. This scenario assumes, in the long term, a continuous rising of greenhouse gas emissions, low penetration of renewable energy sources, intensive use of coal, and high population growth.

The changes in the PV power potential were calculated considering changes in the solar irradiance, air temperature, and wind speed towards the end of the century (2070-2099) with respect to the reference period (1970-1999). We considered a 30-years period because it is a long enough projection to scale the lifetime of PV modules [25]. For each climate scenario (RCP2.6, RCP8.5, and historical), we computed the mean of the six climate change models as proposed in [8].

It is worth noting that climate models depend on physical parameterization schemes. Table 2 summarizes the key physical parameterization schemes used in the regional models used in this paper. To deal with the uncertainty caused by different parameterization schemes, it is recommended to work with the biggest possible ensemble of climate models. In this paper, we used all the climate models available for the study region. These models cover just two climate scenarios: RCP2.6, and RCP8.5 [56]. With these models, we evaluated the possible change in the photovoltaic power potential according to these extreme climate change scenarios.

**Table 2:** Physical parameterization schemes of the RCP models.

	Physical parameterization schemes	Reference
GERICS-REMO2015	Soil processes heat transfer	[26]
	Vertical diffusion and surface fluxes	[27]
	Radiation	[28], [29]
	Stratiform clouds	[30], [31]
	Cumulus convection	[32], [33]
	Fractional surface cover	[34]
	Freezing and thawing of soil water	[34]
	Monthly variation of vegetation parameters	[35]
	Snow-free land surface albedo	[35]

ICTP-RegCM4-7	Radiation	[36], [37]
	Land surface	[38], [39]
	Planetary boundary layer	[40], [41]
	Convective precipitation	[42], [43], [44], [45]
	Large-scale precipitation	[46]
	Cloud microphysics	[47], [48], [49]
	Ocean flux	[50]
	Prognostic sea surface skin temperature	[51]
	Pressure gradient	[52]
	Lake model	[53]
	Aerosols and dust	[54], [55]

## 3 Results

The maps presented in this paper were constructed using Tableau. This software uses Mercator projection for map construction. The spatial resolution of the database is 25 km in both latitude and longitude. Therefore, each pixel on a map represents a 25 x 25 km area.

### 3.1 Change in solar irradiance and its influence on PV power potential

**Figure 3:**
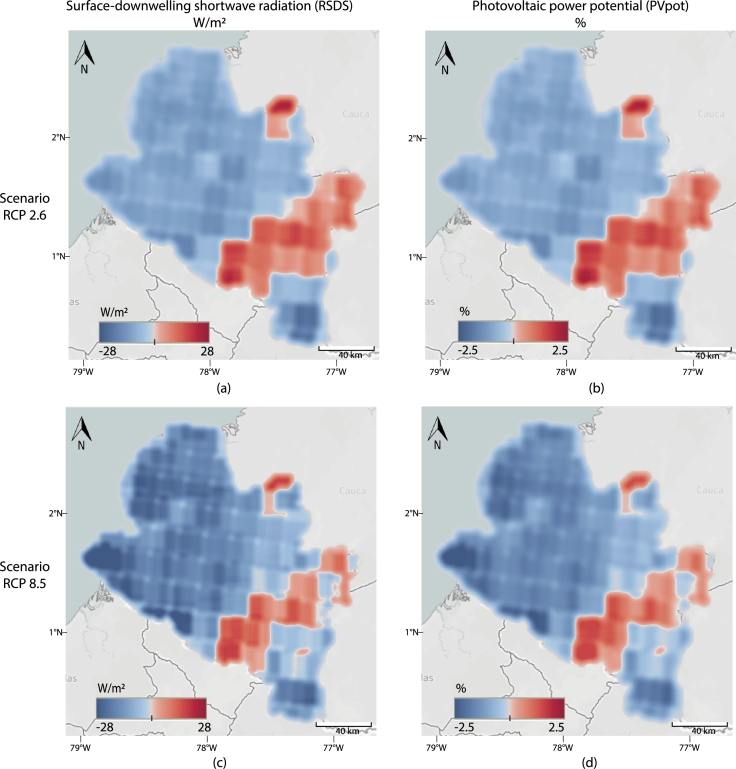
**Solar irradiance change analysis.** The left column shows the change in solar irradiance, in Watts per square meter, towards the end of the century (2070-2099) with respect to the reference period (1970-1999). The right column represents the respective change in PV power potential in percentage. In particular, (a) Changes in RSDS with RCP2.6, (b) *PV*_*pot*_ with RCP2.6, (c) RSDS with RCP8.5, and (d) *PV*_*pot*_ with RCP8.5.

Figs. 3(a) and 3(b) show the solar irradiance changes in the reference period (1970-1999) compared to the end of the century (2070-2099) and its effects on the PV power potential for the whole Nariño territory, according to the RCP2.6 scenario. There is a general lessening in solar irradiance, especially in the Pacific region, with a decrease of up to $17\text{ W}/{\text{m}}^{2}$. Few areas do not show significant differences, and the Andean region shows irradiance increase with values up to $22\text{ W}/{\text{m}}^{2}$. These solar irradiance variations do not have a considerable impact on PV power potential, causing maximum decreases of 1.5% in the northwest, and even increase up to 2% is observed in the central zone of Nariño.

The effects of the RCP8.5 scenario on the PV power potential for the whole Nariño territory are shown in Figs. 3(c) and 3(d). In this case, the solar irradiance presents a maximum decrease of up to $28\text{ W}/{\text{m}}^{2}$, whereas the maximum increase is $26\text{ W}/{\text{m}}^{2}$. Similar to the RCP2.6 scenario, the Pacific region shows a general lessening in solar irradiance, whereas the Andean region presents an overall increase. These variations in solar irradiance according to the RCP8.5 scenario have a slightly larger impact on the PV potential, causing maximum diminutions of 2.5% and an increase of up to 2.5%, respectively in the northwest and central zone of Nariño.

### 3.2 Change in temperature and its influence on PV power potential

**Figure 4:**
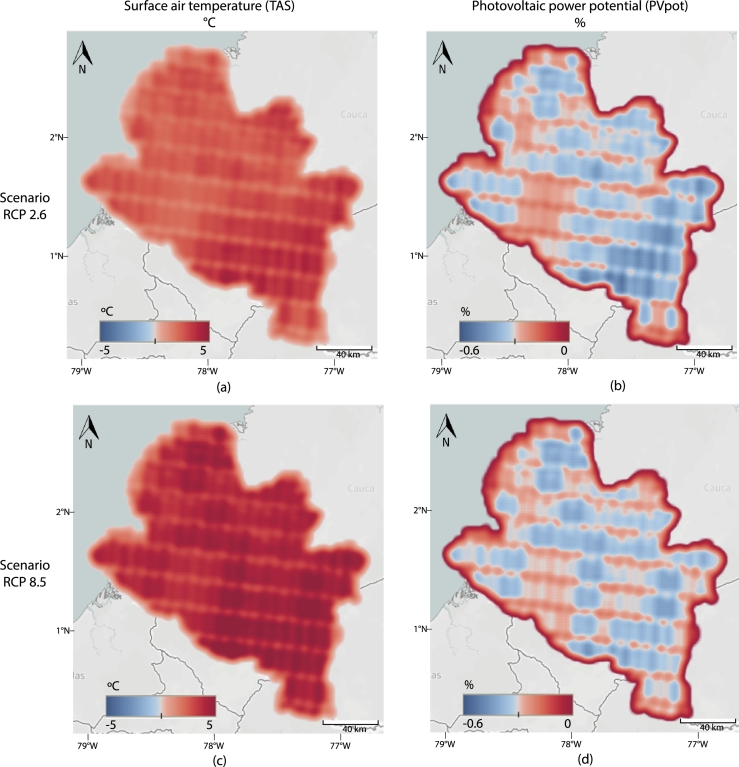
**Temperature change analysis.** The left column shows the change in temperature, in degrees Celsius, towards the end of the century (2070-2099) with respect to the reference period (1970-1999). The right column represents the respective change in PV power potential in percentage. In particular, (a) Changes in TAS with RCP2.6, (b) *PV*_*pot*_ with RCP2.6, (c) TAS with RCP8.5, and (d) *PV*_*pot*_ with RCP8.5.

Figs. 4(a) and 4(b) show the air temperature change in the end of the century (2070-2099) with respect to the reference period (1970-1999) and its effects on the PV power potential according to the RCP2.6 scenario. There is a general increase in the air temperature, especially in the Andean region, with a maximum of 2C∘. The northwestern side of Nariño shows an increase of approximately 1C∘, and the minimum increase in the whole territory is approximately 0.6C∘. These changes can affect the PV power potential with a 0.25% decrease in PV cell performance in warmer locations. However, few regions do not show a significant impact.

According to the RCP8.5 scenario, the change in air temperature and its effects on the PV power potential are shown in Figs. 4(c) and 4(d), respectively. In this case, the changes are more noticeable, with a minimum increase of 2.5C∘ and a maximum of 5C∘. The most affected region is the southeast, where the PV power potential could decrease up to 0.6%.

### 3.3 Change in wind speed and its influence on PV power potential

**Figure 5:**
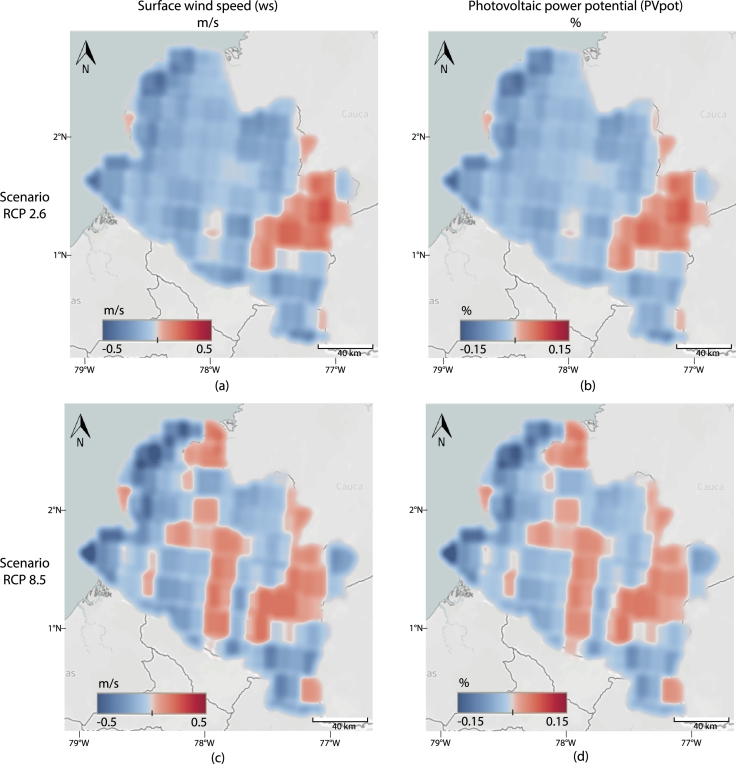
**Wind change analysis.** The left column shows the change in wind speed, in meters per second, towards the end of the century (2070-2099) with respect to the reference period (1970-1999). The right column represents the respective change in PV power potential in percentage. In particular, (a) Changes in WS with RCP2.6, (b) *PV*_*pot*_ with RCP2.6, (c) WS with RCP8.5, and (d) *PV*_*pot*_ with RCP8.5.

The projected changes in wind speed with the RCP2.6 scenario and its influence on the PV potential are shown in Figs. 5(a) and 5(b). In general, the wind speed remains almost constant, with changes varying between −0.2 m/s to 0.16 m/s. For the RCP8.5 scenario, the results are presented in Figs. 5(c) and 5(d). In this case, changes in wind speed vary between −0.5 m/s to 0.2 m/s. In both cases, the change in $PV_{pot}$ is less than 0.15%.

## 4 Discussion

### 4.1 Change in solar irradiance and its influence on PV power potential

According to our results, the variable that will most affect PV potential in Nariño at the end of the century is RSDS, generating a maximum decrease of 2.5% and a maximum increase of 2.5% on $PV_{pot}$ in the RCP8.5 scenario, and 1.5% and 2% in the RCP2.6 scenario. These changes in RSDS will affect the Pacific region, in the northwest of Nariño, with an overall decrease of about 1.6% on $PV_{pot}$. However, this reduction can be considered small enough to threaten the future of PV solutions in this region. Therefore, we consider that PV systems are feasible solutions as electric power sources for the Pacific region, mainly because this territory, which belongs to the non-interconnected zone, has lush vegetation that makes its integration into the interconnected system difficult.

On the other hand, for the Andean region, located at the center and southeast Nariño, changes in RSDS are expected to increase the $PV_{pot}$ by an average of 1% for both RC8.5 and RCP2.6 scenarios. This region is the most populated of Nariño, and it is characterized by its mountainous landscape. Although this zone is part of the national interconnected system, it has electricity supply problems due to the fact that it is at the tail end of the national grid. Also, the power supply depends mainly on hydroelectric plants, which are vulnerable to meteorological phenomena such as El Niño and La Niña, which have already caused energy rationing [57]. Therefore, it is necessary a transition from hydroelectric plants to other non-conventional renewable energies, especially PV systems that have a great potential in this region.

### 4.2 Change in temperature and its influence on PV power potential

The air temperature is the second variable that most affects PV potential. $PV_{pot}$ will have a maximum decrease of 0.6% and 0.25% for RCP8.5 and RCP2.6, respectively. In this case, the Andean region is the most affected one, while the Pacific region is the less affected zone. Although temperature changes are expected to decrease $PV_{pot}$ in most of the Nariño territory, these changes are less than 1%. Therefore, the photovoltaic potential for this region is still positive.

Also, it is important to point out that an increase above 4C∘ in Nariño would be above the upper bound of 2C∘ in 2050 defined in [58], [59], which could cause harmful consequences for nature. Note the relationship between the changes in temperature and the physiographic regions of Nariño, shown in Fig. 1. The mountainous regions show a stronger increase in temperature than the one observed in the Pacific region.

### 4.3 Change in wind speed and its influence on PV power potential

Finally, in both climate change scenarios, changes of wind speed are too small to significantly affect the PV power potential. Therefore, the influence of wind speed on the PV potential can be considered negligible for the studied region. These results of the slight impact of wind speed on the PV power potential are in accordance with the results presented in [8], [11], [12], [14], [60].

## 5 Conclusion

We have studied the impact of climate change on PV power potential, in the region of Nariño, Colombia, towards the end of the century, due to two climate change scenarios based on the CORDEX-CORE framework, one optimistic and the other pessimistic. We found that solar irradiance, temperature, and wind speed changes will cause a maximum decrease of around 2.5%, with some unaffected areas, and a maximum increase of 2.5% on the PV power potential, with respect to the pessimistic climate change scenario. According to the National Renewable Energy Laboratory (NREL), photovoltaic solar panels are expected to continue increasing their efficiency and lifespan while reducing their cost. Also, local governments are developing policies to support the PV system deployment. Such a scenario suggests that a reduction of 2.5% in some specific regions of Nariño is not a potential threat to the development of PV systems in the region. Therefore, with these small expected changes in PV potential, it is unlikely that, by the end of the century, the PV sector in Nariño will be significantly affected.

This work laid an important foundation for studying the feasibility of implementing renewable energy systems in a Colombian region that belongs to the non-interconnected zones.

**Table:** 

Abbreviation	Definition
CORDEX	Coordinated Regional Downscaling Experiment
CORE	Coordinated Output for Regional Evaluations
GCM	Global climate models
PV	Photovoltaic
PVpot	Photovoltaic power potential
RCM	Regional climate model
RCP	Representative concentration pathway
RSDS	Surface-downwelling shortwave radiation
TAS	Surface air temperature
WS	Surface wind speed

## Declarations

### Author contribution statement

Gabriel Narvaez, Luis Felipe Giraldo: Conceived and designed the experiments; Performed the experiments; Analyzed and interpreted the data; Contributed reagents, materials, analysis tools or data; Wrote the paper.

Michael Bressan, Andres Pantoja: Contributed reagents, materials, analysis tools or data; Wrote the paper.

### Funding statement

Andres Pantoja was supported by Ministerio de Ciencia, Tecnología, e Innovación de Colombia Minciencias 65472. Gabriel Narvaez was supported by 10.13039/100017703Fundación CeiBA. Luis Felipe Giraldo was supported by Nvidia GPU Grant program and 10.13039/501100006070Universidad de los Andes, Colombia Convocatoria para proyectos con el alcance de los objetivos de desarrollo sostenible and Vice Presidency for Research & Creation at Universidad de los Andes, Colombia Publication Fun.

### Data availability statement

Data included in article/supp. material/referenced in article.

### Declaration of interests statement

The authors declare no conflict of interest.

### Additional information

No additional information is available for this paper.

## Figures and Tables

**Figure 6:**
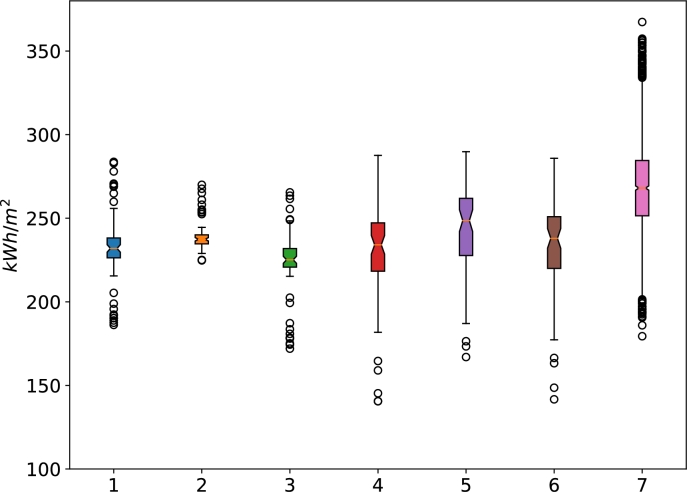
Boxplot of the solar irradiance by the climate change models and the NREL database. The six first boxplots represent the climate models in the same order as in Table 2, the last boxplot represents the NREL database.

## Appendix A

### A.1 PV power potential formulation

The PV power potential can be expressed as a dimensionless magnitude that accounts for the performance of PV cells related to environmental conditions and its nominal power capacity. Solar radiation and air temperature directly affect the performance of PV cells [61]. While higher solar radiation implies better PV performance, higher PV-cell temperature, which is affected by air temperature, solar radiation, and other physical parameters, reduces the potential. For this study, we consider monocrystalline silicon panels, which have better efficiency and are widely used in the industry [62]. According to [8], the PV power potential $PV_{pot}$ can be expressed as

(1) $$PV_{pot}=PR\frac{RSDS}{RSDS_{STC}},$$

where $RSDS_{STC}=1000\text{ W}/{\text{m}}^{2}$ with STC for standard test conditions. RSDS is the surface-downwelling shortwave radiation, and *PR* is the performance ratio, which depends on the PV cells efficiency due to changes in their temperature. This ratio is defined as

(2) $$PR=1+\gamma (T_{cell}-T_{STC}),$$

where γ=−0.005C−1∘ is a constant that depends on physical parameters of PV cells [61], and TSTC=25C∘. The cell temperature $T_{cell}$ can be described by

(3) $$T_{cell}=c_{1}+c_{2}TAS+c_{3}RSDS+c_{4}WS,$$

with c1=4.3C∘, $c_{2}=0.943$, and c3=0.028C∘m2/W, c4=−1.528C∘s/m being constants that depend on physical parameters of the PV cells [61]. In this model, *TAS* is the surface air temperature, and *WS* is the surface wind speed. According to (1) and (2), if environmental conditions are equal to standard conditions, $PV_{pot}=1$ and PV power output reaches the nominal value. If $T_{cell}>25$ and/or RSDS<1000, $PV_{pot}<1$ and the PV power output will be less than the rated power of the module. On the other hand, if $T_{cell}<25$ and/or RSDS>1000, the PV panel will improve the nominal generation with a $PV_{pot}t>1$.

Using Equations (1) - (3) $PV_{pot}$ can be expressed as:

(4) $$PV_{pot}=RSDS(a+bRSDS+cTAS+dWS),$$

where

$$a=\frac{1+\gamma (c_{1}-T_{STC})}{RSDS_{STC}},b=\frac{\gamma c_{3}}{RSDS_{STC}},c=\frac{\gamma c_{2}}{RSDS_{STC}},d=\frac{\gamma c_{4}}{RSDS_{STC}}.$$

From Equation (4), changes in $PV_{pot}$, denoted as $\Delta PV_{pot}$, can be calculated as:

(5) $$\Delta PV_{pot}=\;\Delta RSDS\left(a+b\Delta RSDS+2bRSDS+cTAS+dWS\right)+cRSDS\cdot \Delta TAS+dRSDS\cdot \Delta WS+c\Delta RSDS\cdot \Delta TAS+d\Delta RSDS\cdot \Delta WS$$

From Equation (5), the changes in $PV_{pot}$ due to the individual contributions of ΔRSDS, ΔTAS, and Δ*WS* can be calculated. For example, the estimated change in $PV_{pot}$ due to changes in *TAS* is given by taking ΔRSDS=ΔWS=0 and keeping the remaining variables (RSDS, *WS*) constant at their annual mean for the reference period (1970-1999). This methodology is the same as that presented in [8], [14]. Observe that it is not possible to fully isolate the contribution of each variable due to the cross-products in the last two terms of Equation (5).

### A.2 Data validation

The simulation of clouds in climate models is challenging. As it is done in [63] and [64], one approach to verify the bias in cloudiness in the region is to verify the accuracy of the climate models with respect to local measurements. However, there are just a few meteorological stations in the region that are widely spread. Therefore, we used satellite information from the National Solar Radiation Database (NREL) and compared both databases. We verified the ability of the climate models to represent solar irradiance. As depicted in Fig. 6, the boxplot shows how statistically the models differ. The first six boxplots correspond to the climate models, whereas the last one represents the NREL data. On average, the six climate change models present a radiation value of $233\text{ W}/{\text{m}}^{2}$, while the NREL data show an average of $268\text{ W}/{\text{m}}^{2}$ for the region. In general, the climate models tend to show lower solar radiation. However, this is still a good approximation of the behavior of solar radiation in Nariño.

## Footnotes

1 Fig. 1 used *Narino Topographic* by Dr Brains (https://commons.wikimedia.org/wiki/File:Narino_Topographic_2.png?uselang=en) and *Narino in Colombia (mainland)* by TUBS (https://commons.wikimedia.org/wiki/File:Narino_in_Colombia_(mainland).svg), which are licensed under CC BY-SA 3.0 (https://creativecommons.org/licenses/by-sa/3.0/deed.en). The second figure was put on top of the first one and an elevation scale was added.
